# Clinical and histopathological characterization of enfortumab vedotin-associated cutaneous toxicities: A case series

**DOI:** 10.1016/j.jdcr.2024.12.019

**Published:** 2024-12-30

**Authors:** Ista A. Egbeto, Evangelia Vlachou, Daniela Barata Herrera, Stephanie Russell, Joel C. Sunshine, Jean Hoffman-Censits, Sima Rozati

**Affiliations:** a Department of Dermatology, Johns Hopkins University School of Medicine, Baltimore, Maryland; b Department of Urology, The Johns Hopkins Greenberg Bladder Cancer Institute, Baltimore, Maryland; c Department of Oncology, Johns Hopkins University Sidney Kimmel Comprehensive Cancer Center, Baltimore, Maryland

**Keywords:** AGEP, drug eruption, enfortumab vedotin, oncodermatology, oncology

## Introduction

Locally advanced (la) or metastatic urothelial cancer (mUC) is an aggressive disease with a median overall survival (OS) of 13 months with platinum-based chemotherapy which was the standard of care until recently.

Enfortumab vedotin (EV) is a recently approved antibody-drug conjugate (ADC) consisting of a human IgG1 monoclonal antibody targeting NECTIN-4, linked to monomethyl auristatin E (MMAE), a microtubule-disrupting agent.^1^, ^2^, ^3^ EV has revolutionized the management of la/mUC by demonstrating nearly double median OS in combination with pembrolizumab versus platinum-based chemotherapy in a phase III trial.^4^ As a result, EV in combination with pembrolizumab received Food and Drug Administration approval as a first-line treatment for patients with locally advanced or metastatic urothelial cancer (la/mUC). Following Food and Drug Administration approval, there has been a significant uptake of EV plus pembrolizumab as the first-line treatment.^5^ Even as monotherapy, EV shows promising results. The phase III EV-301 trial revealed that EV led to a median OS of 12.9 months and a median progression-free survival (PFS) of 5.6 months, in contrast to 9.0 months and 3.7 months, respectively, with late-line chemotherapy.^6^

EV 1.25 mg/kg is administered intravenously on days 1, 8, and 15 of a 28-day cycle for monotherapy and on days 1 and 8 for combination therapy with pembrolizumab.^4^ All EV doses are capped at 125 mg. During the trials, cutaneous toxicities were one of the most common adverse events that led to dose reduction, interruption, or treatment discontinuation. In the EV-301 trial, treatment-related rash occurred in 43.9% of participants.^6^ Within the safety data for 680 patients from trials involving EV as a single agent, 55% of those treated with 1.25 mg/kg dose experienced skin reactions, most frequently maculopapular rash (23%) and pruritus (33%). Severe skin reactions of grade 3 or higher were reported in 13% of patients, including maculopapular rash, erythematous rash, drug eruption, eruption favoring intertriginous areas such as symmetrical drug-related intertriginous and flexural exanthema, bullous dermatitis, exfoliative dermatitis, palmoplantar erythrodysesthesia, and Stevens-Johnson syndrome (SJS) or toxic epidermal necrolysis (TEN)-like cutaneous eruption without mouth ulceration.^3^ The median time to onset of severe skin reactions was 0.6 months.^6^ The reported incidence of skin events is higher in combination trials with pembrolizumab. Phase 2 studies show 67% of patients experiencing skin reactions were treated with EV and/or pembrolizumab dose reduction, interruption, and/or corticosteroids with most cases (36%) treated with discontinuation of either EV or pembrolizumab.^7^^,^^8^ EV-302 reported 35% of patients experiencing maculopapular rash and 40% experiencing pruritus.^4^^,^^9^

The mechanism behind EV-related skin toxicity involves the delivery of MMAE into normal tissues expressing Nectin-4, which is found in the epidermis and in skin appendages such as sweat glands and hair follicles, explaining the appearance of the rash in the intertriginous areas. This selectivity makes epidermal keratinocytes susceptible to the antimitotic effects of MMAE, which disrupts microtubule networks, resulting in apoptotic death.^1^^,^^3^^,^^10^ This retrospective series attempts to provide a detailed clinical description of EV-related skin toxicities compared to what is commonly reported in clinical trials and correlate them with histological findings to facilitate the identification and management of these reactions in patients undergoing treatment.

## Methods

A retrospective review of EV-treated patients at Johns Hopkins who required a skin biopsy due to EV-related cutaneous toxicity was performed. All patients were evaluated by a dermatologist, who also performed the skin biopsy, which was read by one dermatopathologist at Johns Hopkins University.

Medical record review included all medication exposures prior to EV initiation until the date of rash onset, clinical presentation, distribution, and morphology of the rash, response to EV treatment, and survival data. The time to onset of the rash was documented as the number of days from the initial EV dose to the patient-reported appearance of the skin eruption.

Descriptive statistics were used to summarize patient characteristics. We calculated OS and PFS for each patient and mean OS and PFS for the entire cohort. Statistical analysis was performed using R 4.2.2 and Microsoft Excel.

The Johns Hopkins University Institutional Review Board approved this study, and all patients gave written consent. Authors had access to identifiable data to review notes and images for data collection. The study conformed to the case series reporting standards.

## Results

### Baseline characteristics

From December 2017 to April 2024, we identified 7 patients treated with EV who had skin biopsies due to EV-related cutaneous eruptions. In our cohort, 5/7 (71.4%) received EV monotherapy, and 2/7 (28.6%) received EV with pembrolizumab. Four patients received pembrolizumab before EV, with an average interval of 32.25 days between therapies. One patient initially received EV monotherapy and later switched to combination therapy after disease progression.

Patient 3 was excluded as they were on dual therapy with EV+ pembrolizumab, and their rash remained stable during EV monotherapy while receiving systemic and topical steroids at a reduced dose, which improves EV tolerability. Due to overlapping toxicities, it is challenging to attribute effects to a specific therapy. EV was used as the first, second, and >third line of therapy in 2 (28.6%), 3 (42.9%), and 2 (28.6%) patients, respectively. All started EV at full dose.

Two patients were female (28.6%), and five were male (71.4%). The median age was 76 years (range: 59-83). At EV initiation, Eastern Cooperative Oncology Group Performance Status was 0 for 3 (42.9%) patients and 1 for 4 (57.1%) patients. The primary tumor location was the upper urinary tract for 4 (57.1%) patients, and one patient had squamous cell carcinoma without urothelial cancer. Two patients (28.6%) had a history of psoriasis.

### Response to EV treatment

The best radiographic response to EV was a partial response for 5/6 (83.3%) patients, with one patient refusing radiographic evaluation (Table I). The median PFS in our cohort was 6.83 months (95% confidence interval: 5.45, not reported). The median OS was 13.4 months (95% confidence interval: 6.87, not reported) and 2 patients remain alive. The median duration of treatment was 5.3 months (range: 0.2-7). One patient (14.3%) continued EV after 4 cycles (5.4 months) of therapy.

**Table I:** Baseline characteristics and treatment response

Pt	1	2	3	4	5	6	7
Age at EV initiation	66	83	76	67	76	59	82
Sex	Male	Male	Female	Female	Male	Male	Male
ECOG PS at EV initiation	0	1	1	1	0	1	1
Regimen	EV	EV	EV + P	EV	EV	EV	EV + P
EV line	3	2	1	3	2	3	1
Prior ICI	Yes	Yes	No	Yes	No	Yes	No
ICI timeframe prior to EV (in days)∗	34	48	NA	18	NA	29	NA
Tumor histology	UC	UC	UC	Squamous cell carcinoma	UC	UC w/focal glandular differentiation	UC
Primary tumor location	Bladder	Bladder	Upper tract	Upper tract	Upper tract	Bladder	Upper tract
Metastatic sites	Lung	Lung	Bones	Retroperitoneum	Peritoneum	Liver, bone	Liver, lung
Total number of EV cycles	1	2	5	8	4	5	8
Best physician-assessed radiographic response	Refused imaging	PR	PR	PR	PR	PR	PR

*EV*, Enfortumab vedotin; *ECOG PS*, Eastern Cooperative Oncology Group Performance Status; *ICI*, immune checkpoint inhibitor; *P*, pembrolizumab; *PR*, partial response; *UC*, urothelial carcinoma.

∗ ICI timeframe is calculated as the time between the completion of pembrolizumab therapy and the start of EV.

### EV-related cutaneous events

All patients developed dermatologic toxicity related to EV within the first or second cycle. In 4/6 patients (66.7%), the rash appeared within the first cycle. Three patients (50%) developed skin toxicity after 2 doses of EV, and 1 patient (16.7%) developed a rash after the third dose. Two patients (33.3%) experienced skin toxicities within the second cycle, with both developing rashes after 5 doses (post-C2D8). The time to rash onset varied, with a median of 27.8 days (range: 7-52 days).

Using the Common Terminology Criteria for Adverse Events, 2 patients (33.3%) experienced grade 4 cutaneous adverse events (CAEs), 3 patients (50%) had grade 3 skin toxicities, and one patient (16.7%) had grade 1 skin toxicity.

No patients in this study developed life-threatening reactions such as SJS, TEN, or Drug Reaction with Eosinophilia and Systemic Symptoms-like toxicity. One patient developed numerous nonfollicular pustules on a background of erythema on his trunk, extremities, and intertriginous areas.

**Fig 1:**
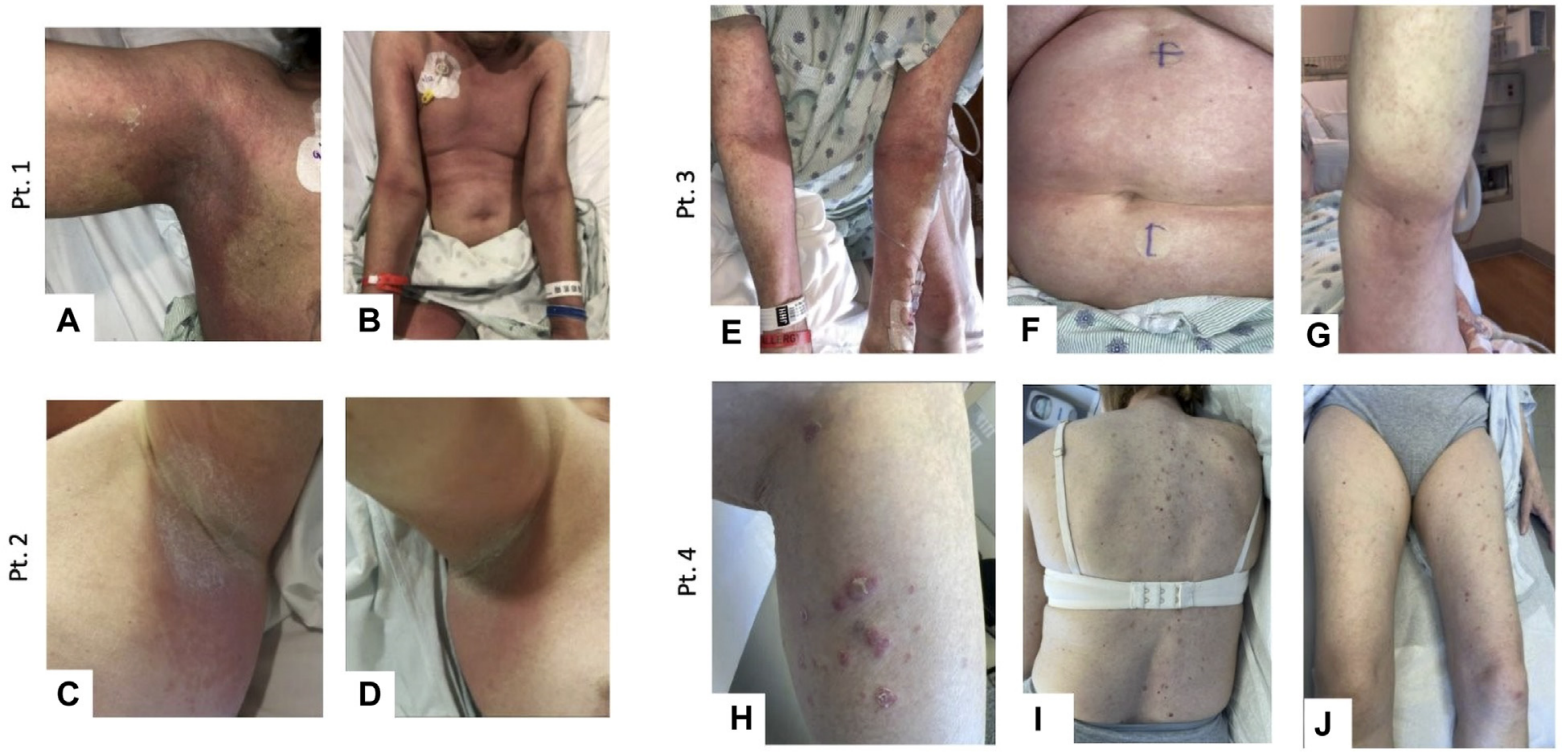
Clinical images of cutaneous eruptions for patients 1-4. **Pt. 1**, Ill-defined erythematous papules in right axilla coalescing into plaques with mild overlying scale (**A**) and diffuse erythematous plaques on trunk and antecubital fossae (**B**). **Pt. 2**, Erythematous macules and plaques with maceration in left axilla (**C**) and right axilla (**D**). **Pt. 3**, Erythematous papules coalescing into plaques on ventral arms and flexures (**E**) and erythematous plaque studding with minute clear fluid-filled vesicles on the posterior thighs (**G**); diffuse erythematous patch on abdomen (**F**). **Pt. 4**, Violaceous scaly papules on left leg (**H**), back (**I**), and bilateral anterior thighs (**J**). *Pt*, Patient.

**Fig 2:**
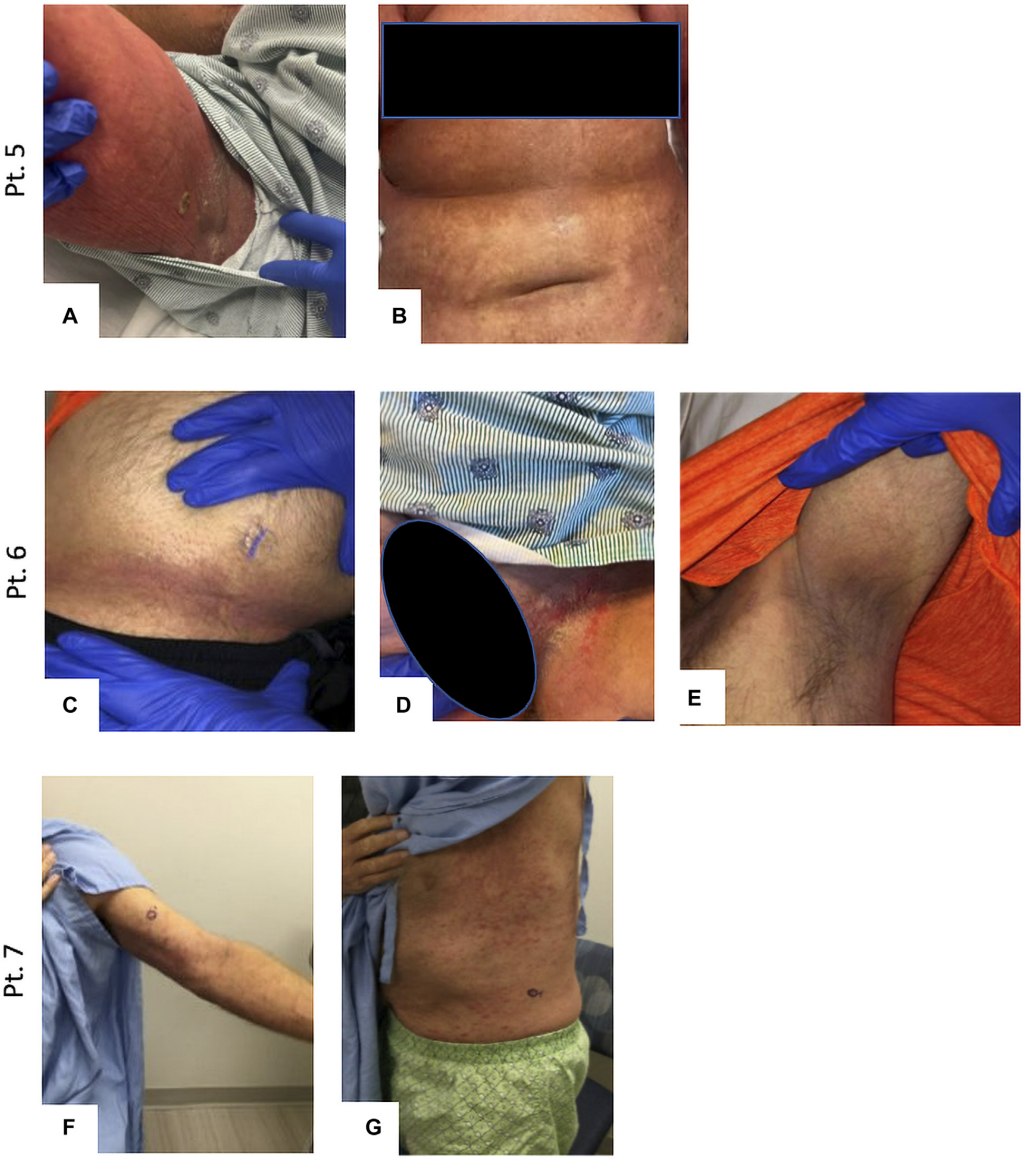
Clinical images of cutaneous eruptions for patients 5-7. **Pt. 5**, Erythematous plaque with maceration in right axilla (**A**); diffuse widespread nearly confluent erythematous macules/patch on trunk (**B**). **Pt. 6**, Erythematous patches in infrapannus region (**C**); significant scrotal edema with erythema and scale in intertriginous areas of groin (**D**), ill-defined erythematous patches in left axilla (**E**). **Pt. 7**, Polymorphous maculopapular eruption with scaling on left arm (**F**); polymorphous maculopapular eruption with scaling across trunk (**G**).

The observed skin toxicities showed overlapping morphologies (Table II, Figs 1 and 2), including (1) lichenoid dermatitis (1/6, 16.7%); and (2) broad drug eruptions (5/6, 83.3%), which encompassed pustular drug eruption (1/6, 16.7%), drug eruption predominantly affecting intertriginous areas (3/6, 50%), and other nonspecific drug eruptions (1/6, 16.7%).

**Table II:** Dermatologic information

Pt	1	2	3	4	5	6	7
Days from start of EV to rash onset	10	52	8	28	7	15	27
Number of EV doses prior to rash	2	5	2	3	2	2	5
Clinical diagnosis	Drug eruption	Drug eruption	Morbilliform drug eruption	Lichenoid drug eruption	Pustular drug eruption	Drug eruption	Drug eruption
Distribution of cutaneous eruption	Axilla, posterior neck, back, bilateral inguinal folds, upper thighs, and lower legs.	Bilateral axilla, inguinal folds, and inframammary folds.	Neck, chest, breasts, shoulders, ventral arms, thighs. Relative sparing of inframammary folds and axillary vaults	Bilateral upper, lower extremities, and back.	Trunk, axilla, groin, and upper extremities.	Axillae, antecubital/popliteal fossae, bilateral auricular region, forehead, and scrotum.	Trunk and extremities
Grade of rash	4	4	3	1	3	3	3
Treatment	Systemic + topical steroids	Systemic+topical steroids	Systemic + topical steroids	Topical steroids + oral antihistamines	Systemic + topical steroids	Topical steroids	Systemic + topical steroids
Treatment duration (in days)	45	17	42	120	21	14	180

*EV*, Enfortumab vedotin.

The anatomical distribution was also variable, with 5 patients (83.3%) having multifocal involvement (>4 regions) and 1 patient (16.7%) having localized dermatitis (2 locations). The most common regions involved were the intertriginous areas including the axillae and groin, which was observed for all grade 4 and 2 out of 3 grade 3 CAEs.

**Fig 3:**
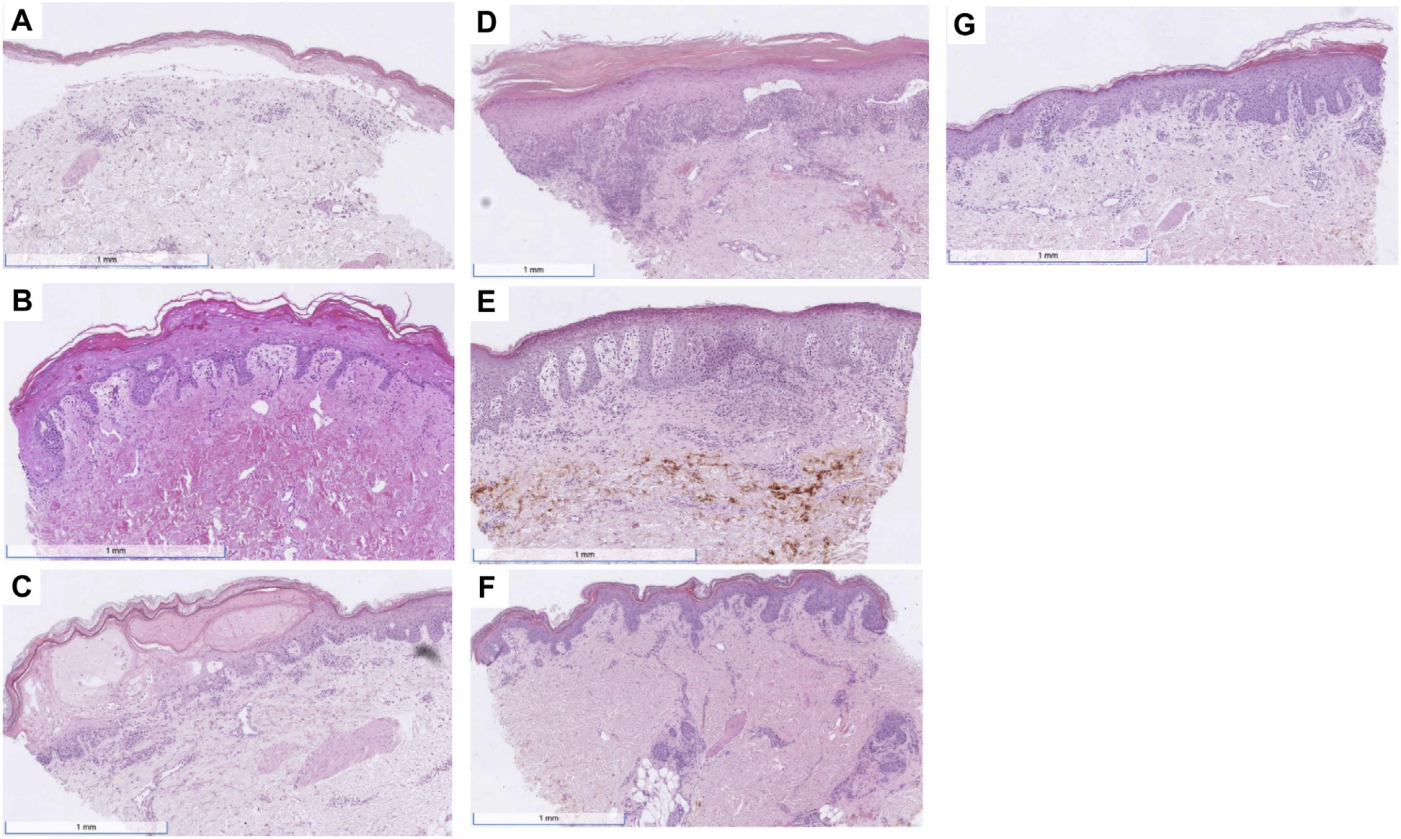
Histology images of the cutaneous eruptions, patients 1-7. **A, Pt. 1**, Interface dermatitis, epidermal dysmaturation, and prominent eosinophils, consistent with drug eruption. The section shows dyskeratotic and bizarrely shaped keratinocytes along with subepidermal separation. There is a sparse superficial perivascular infiltrate of lymphocytes and many eosinophils. **B, Pt. 2**, Interface dermatitis, consistent with drug eruption. There are ring mitoses with pyknotic keratinocytes in the epidermis. **C, Pt. 3**, Spongiotic and vacuolar interface dermatitis with eosinophils, favor drug eruption. The section shows skin with focal parakeratosis. The epidermis is spongiotic and contains occasional lymphocytes. The basal layer focally displays vacuolar alteration, which is associated with a sparse lymphocytic infiltrate at the dermal epidermal junction and dyskeratotic keratinocytes. A superficial perivascular infiltrate is also present that is predominantly lymphocytic. There are ring mitoses with pyknotic keratinocytes in the epidermis. **D, Pt. 4**, Lichenoid interface dermatitis c/w lichenoid drug eruption. Examination of the biopsy shows hyperkeratosis, wedge-shaped hypergranulosis, acanthosis with saw-toothed rete ridges, and degeneration of basal keratinocytes with scattered dyskeratotic cells at the dermal-epidermal junction. The dermis demonstrates a band-like superficial infiltrate composed of lymphocytes, histiocytes, and occasional melanophages. **E, Pt. 5**, Subcorneal pustular dermatitis consistent with pustular drug eruption. **F, Pt. 6**, Vacuolar interface dermatitis with dyskeratosis consistent with a drug reaction. The section shows hyperkeratosis, subtle epidermal dysmaturation, and vacuolar changes of basal keratinocytes with lymphocytic cytosis and dyskeratotic cells along the dermal-epidermal junction and above it. The dermis demonstrates a scant perivascular lymphohistiocytic inflammatory infiltrate. Mild intraepidermal ring mitoses are noted. **G, Pt. 7**, Mild vacuolar interface dermatitis with mounds of parakeratosis. The biopsy shows hyperkeratosis, mounds of parakeratosis, subtle vacuolar changes of basal keratinocytes, occasional dyskeratotic cells along the dermal-epidermal junction, and lymphocytic exocytosis. The dermis demonstrates a mildly dense superficial perivascular infiltrate composed of lymphocytes and histiocytes. Intraepidermal ring mitoses are noted.

Skin biopsy specimens demonstrated predominantly vacuolar interface dermatitis with spongiosis, dyskeratosis, and the presence of dermal eosinophils (Fig 3). A skin biopsy from patient 4 showed subcorneal neutrophils with epidermal dysmaturation. Patients 2, 6, and 7 (3/6) had chemotherapy-induced histological changes including nuclear atypia, basal layer squamatization, and arrested intraepithelial ring mitoses. Patient 3 also showed ring mitoses but was omitted from our analysis. Direct immunofluorescence was negative for all specimens.

## Management

Four patients (66.7%) were admitted for a new onset of rash. Three patients (50%) had other reasons for admission, including hypotension, neutropenia, and diarrhea. Two patients with grade 4 CAEs were treated with systemic steroids (0.5-1 mg/kg/day) for an average of 31 days, along with topical triamcinolone 0.1% ointment. The average duration of systemic steroid treatment was 71.6 days. The patient with grade 1 skin toxicity was treated exclusively with topical clobetasol 0.05% ointment.

All patients initially experienced improvement in their rash following treatments. Two patients (28.6%) discontinued EV due to CAEs. After tapering systemic steroids, one patient experienced a recurrence of the rash, which resolved with topical triamcinolone 0.1% ointment.

## Discussion

EV, an ADC, has been efficient in managing locally advanced or metastatic urothelial cancer (la/mUC), alone and with pembrolizumab.

Investigating the onset and morphology of EV-related CAEs, our cohort developed skin toxicities within the first or second cycle of EV, with a median time to rash onset of 27.8 days. Therefore, we recommend regular and frequent monitoring for dermatologic events throughout treatment, starting at the first cycle.^3^ Of note, 5/6 (83.3%) of our cohort received immunotherapy before or with EV, which has been reported as contributing to the severity of CAEs.^11^

83.3% of patients (5/6) showed multifocal or widespread cutaneous involvement, while one patient presented with localized dermatitis on the extremities. The most affected anatomical regions were the intertriginous zones resembling toxic erythema of chemotherapy (TEC) and symmetrical drug-related intertriginous and flexural exanthem. None of the patients developed SJS, TEN, or Drug Reaction with Eosinophilia and Systemic Symptoms-like eruptions. However, one (case 5) presented with rapid, widespread erythema and pustules in intertriginous areas within 7 days of EV initiation. Histology showed psoriasiform epidermis with subcorneal pustules, suggestive of acute generalized exanthematous pustulosis (AGEP) or generalized pustular psoriasis. Although the patient had a history of psoriasis, no active lesions were found. There was no fever, neutrophilia, or bacterial growth, and the rash resolved with steroids without recurrence after resuming EV at a lower dose. Distinguishing between generalized pustular psoriasis and AGEP is challenging both clinically and histologically, and therefore, neither can be definitively excluded. This rapid pustular eruption has not been previously reported as a skin toxicity associated with EV, making this case potentially a novel association.

In reviewing these 6 cases along with previously reported cases, characteristic histopathologic features of EV drug eruption include keratinocyte dysmaturation accompanied by interface changes, a superficial perivascular infiltrate containing eosinophils, and intraepithelial ring mitoses. Intraepithelial ring mitoses, identified in 3 of our patients have been reported as a defining feature of chemotherapy-induced histologic changes in cutaneous EV toxicity.^12^ Ring mitoses, interface dermatitis, and epidermal dysmaturation, commonly seen in taxane-induced eruptions like TEC, reflect the histopathologic effects of taxanes, which target microtubules and cause mitotic arrest.^13^ Similarly, EV-induced rashes may result from MMAE's antimitotic effects on keratinocytes, as EV binding to cutaneous nectin-4 disrupts keratinocyte microtubules, mimicking the action of taxanes. While squamous metaplasia of eccrine glands was not observed on histology, the presence of ring mitoses in 3 out of 6 patients may suggest a TEC-like eruption.

Clinically, the 3 patients with ring mitoses had grade 4 (1/3) and 3 (2/3) rashes, with varying distributions and morphologies.

Four out of 6 patients (66.7%) received pembrolizumab therapy prior to EV, with an average of 32.25 days between pembrolizumab completion and starting EV. Pembrolizumab, a PD-1 inhibitor, is associated with dermatologic immune-related adverse events such as eczematous or lichenoid rashes, psoriasis, or autoimmune bullous disorders.^14^ Patient 4 developed a grade 1 lichenoid eruption 28 days after starting EV but had received pembrolizumab 18 days earlier. As lichenoid eruptions are a known toxicity of pembrolizumab, the cutaneous eruption may not be solely attributed to EV, and pembrolizumab likely contributed to the presentation.

Our findings underscore the importance of dermatological evaluation in patients undergoing EV treatment, further emphasizing the need for vigilance regarding CAEs, implications for patient quality of life, and potential impact on continuing oncologic treatment. As ADCs like EV become more prevalent, the oncology and dermatology communities must maintain an open dialogue to optimize patient care for managing these emergent toxicities in the inpatient and outpatient setting.

## Conflicts of interest

None disclosed.
